# Complete Genome Sequence of the Novel Klebsiella pneumoniae Phage Marfa

**DOI:** 10.1128/MRA.00748-19

**Published:** 2019-07-18

**Authors:** Laith Harb, Justin Boeckman, Heather Newkirk, Mei Liu, Jason J. Gill, Jolene Ramsey

**Affiliations:** a Center for Phage Technology, Texas A&M University, College Station, Texas, USA; b Department of Animal Science, Texas A&M University, College Station, Texas, USA; DOE Joint Genome Institute

## Abstract

Here, we describe the complete genome sequence of the T4-like Klebsiella pneumoniae myophage Marfa. In its 168,532-bp genome, Marfa has 289 genes, for which 122 gene functions were predicted. Many similar proteins are shared between Marfa and phage T4, as well as its closest phage relatives.

## ANNOUNCEMENT

Carbapenemase-producing Klebsiella pneumoniae (KPC) is a multidrug-resistant enteric bacterium that has emerged as a global health threat (1). KPC is a causative agent of pneumonia and is one of the most common pathogens in hospital-acquired infections, with an overall mortality rate in infected patients between 22% and 59% (2, 3). Reliable therapeutic drugs are not available; therefore, bacteriophage-based therapy is a potential alternative. Here, we describe the novel Klebsiella pneumoniae phage Marfa.

Bacteriophage Marfa was isolated from chloroform-sterilized pooled swine fecal samples from Texas and Michigan. Marfa replicates on a pKpQIL plasmid-cured derivative of K. pneumoniae strain 1776c (4) under aerobic conditions in tryptic soy broth or agar (Difco) at 37°C, and phage propagation was done using the soft agar overlay method (5). Genomic DNA was isolated with the Promega Wizard DNA clean-up kit, according to the protocol from Summer (6) and prepared for sequencing with a TruSeq Nano low-throughput kit. Sequencing occurred on an Illumina MiSeq instrument, with 250-bp paired-end reads. From 382,820 total reads in the index, sequence reads were trimmed using FASTX-Toolkit v0.0.14 (http://hannonlab.cshl.edu/fastx_toolkit/) after quality control with FastQC (www.bioinformatics.babraham.ac.uk/projects/fastqc). Assembly into a single contig at 119-fold coverage was accomplished with SPAdes v3.5.0 using default parameters (7). The contig was confirmed to be complete by PCR (forward primer, 5′-CCTTGCTGGTCCGTGATTT-3′; reverse primer, 5′-CTGGTGGGTCGTGATAAGATG-3′) using outward-facing primers and by Sanger sequencing of the product. Protein-coding and tRNA genes were predicted by GLIMMER v3.0, MetaGeneAnnotator v1.0, and ARAGORN v2.36 (8–10). TransTermHP v2.09 was used to annotate rho-independent terminators (11). All of the tools used for analysis and annotation are available on the Center for Phage Technology Galaxy and Web Apollo instances (https://cpt.tamu.edu/galaxy-public/) (12, 13). Functional annotations used evidence from InterProScan v5.22-61 and BLAST v2.2.31 versus the NCBI nonredundant, UniProtKB, Swiss-Prot, and TrEMBL databases, with a cutoff of 0.001 for the E value (14–16). As needed, TMHMM v2.0 and HHpred with ummiclust30_2018_08 for multiple-sequence alignment (MSA) generation and PDB_mmCIF70 for modeling in the HHsuite v3.0 release provided supplementary evidence (17, 18). Marfa was negatively stained with 2% (wt/vol) uranyl acetate and viewed by transmission electron microscopy at the Texas A&M Microscopy and Imaging Center (19).

Marfa is a myophage with a 168,532-bp genome, a coding density of 94.8%, and a G+C content of 41.0%. The G+C content is less than that of K. pneumoniae, which has an average G+C content of >50%. Genome analysis revealed 289 protein-coding genes, for which 122 gene functions were predicted. Marfa is T4-like, based on similarity to 111 phage T4 proteins, but it also shares 259 similar proteins with Klebsiella myophage vB_Kpn_F48 (GenBank accession number MG746602). Analysis with progressiveMauve demonstrates that Marfa shares 94% sequence identity across 93% of its genome with vB_Kpn_F48 (20). PhageTerm analysis predicts Marfa to have permuted ends and headful packaging, which are expected of T4-like phages (21, 22). Interestingly, the L-shaped tail fiber gene (NCBI accession number QDB71908) has 37% identity and 79% coverage to the L-shaped tail fiber protein of phage T5 (NCBI accession number YP_006961).

### Data availability.

The genome sequence and associated data for phage Marfa were deposited under GenBank accession number MN044033, BioProject accession number PRJNA222858, SRA accession number SRR8869231, and BioSample accession number SAMN11360393.
